# Diversity and heterogeneity of immune states in non-small cell lung cancer and small cell lung cancer

**DOI:** 10.1371/journal.pone.0260988

**Published:** 2021-12-02

**Authors:** Shawn J. Rice, Chandra P. Belani

**Affiliations:** 1 Penn State Cancer Institute, Hershey, PA, United States of America; 2 Department of Medicine, Milton S. Hershey Medical Center, Hershey, PA, United States of America; University of Nebraska Medical Center, UNITED STATES

## Abstract

Blood-based biomarkers including systemic inflammation (SI) indicators or circulating factors (cytokines, chemokines, or growth factors) are associated with a poor prognosis for lung cancer patients. Collectively these biomarkers can predict the immune state of a patient. We wanted to define and compare the immune states of small cell and non-small cell lung cancer patients, in the hopes that the information gained could lead to overall improvements in patient care and outcomes. Specimens and data from 235 patients was utilized, 49 surgically resected non-small cell lung cancer (NSCLC) patients with no evidence of disease (DF), 135 advanced non-small cell lung cancer (NSCLC), 51 small cell lung cancer (SCLC). SI markers neutrophil-to-lymphocyte ratio (NLR), platelet-to-lymphocyte (PLR), systemic inflammation index (SII), and systemic inflammation response index (SIRI) were determined from blood counts. Forty-seven plasma cytokines were measured using a multiplex bead-based assay. Progression-free survival (PFS) and overall survival (OS) were assessed using Kaplan-Meier and Cox Proportional Hazards models. NSCLC patients had significantly high levels of SI markers than SCLC and DF patients, while NLR, PLR and SII were also higher in SCLC than DF patients. SI optimized marker values to differentiate SI value were; 6.04 (NLR), 320 (PLR), 1615 (SII), and 7.3 (SIRI). Elevated levels NLR (p<0.001), PLR (p<0.001), and SII (p = 0.018) were associated with a worse PFS and OS in NSCLC, while none of the markers were associated with PFS in SCLC patients. NSCLC patients with a poor outcome displayed heterogeneous immune states relative to systemic inflammation and circulating IL-6 markers. These groups could be distinguished based on the cytokines IL-8, TNFα, and IL-27. We identified heterogeneity of immune states in SCLC and NSCLC patients and in NSCLC patients with the poorest prognosis. This heterogeneity could be exploited to improve outcomes for these patients.

## Introduction

The two major types of lung cancer are non-small cell lung cancer (NSCLC) and small cell lung cancer (SCLC) that account for 84 and 13% of all lung cancer cases and 5 year survival rates of 24% and 6%, respectively [1]. NSCLC patients with advanced and metastatic disease with actionable mutations have derived clinical benefits from targeted agents, however, treatment options for NSCLC without actionable mutations and SCLC patients have been limited to chemotherapy, but modulation of the immune system with monoclonal antibodies acting as immune checkpoint inhibitors (ICI) has shown great potential for improving outcomes in these patients [2–4]. Durable responses in these patients can be achieved with ICI therapeutics. Many patients still do not respond to these treatments or the disease progresses, highlighting the need for biological markers to assist oncologists in selecting the best treatment option. For example, tumor expression levels of Programmed Death-Ligand 1 (PD-L1) is a common marker used to assign patients to ICI treatment. Many patients, despite elevated PD-L1 expression fail ICI treatment, while approximately 20% of patients with low or no PD-L1 do respond to treatment [5–7]. PD-L1 is a relatively poor marker for predicting activity of an ICI as it has limited sensitivity and specificity for identifying those patients most likely to respond.

The immune system plays a critical role in the outcomes for lung cancer patients. Chronic inflammation is associated with an increased risk of developing metastasis for various cancers including lung cancer [8]. Causes of chronic or systemic inflammation (SI) are varied and can include smoking or tobacco use, alcohol use, obesity, and diet, among others [8]. It has been noted that elevated markers for SI, such as neutrophil-to-lymphocyte ratio (NLR) or platelet-to-lymphocyte ratio (PLR), are associated with reduced progression-free and overall survival in NSCLC and SCLC patients [9–17]. High SI levels was associated with poor outcomes even when immune modulating ICI were used as treatment [14, 18, 19]. Other markers of immune system function such as various cytokines can predict outcomes in lung cancer patients. IL-6 is one commonly studied example. High levels of circulating IL-6 is associated with a worse progression-free and overall survival in NSCLC patients [20–22]. SI and other blood-based markers are easier to evaluate because they are measured and analyzed from peripheral blood and do not require invasive procedures like biopsies, and thus there is no significant additional cost or harm to the patient.

We were interested in understanding how a patients’ immune state, systemic inflammation and circulating factors, related to outcomes in SCLC versus NSCLC. We utilized a dataset of surgically resected patients with no evidence of disease at the time of blood collection to establish optimized widely pertinent marker levels to differentiate SI. We included the following ratios in this work, NLR and PLR, because they are the most studied SI markers. Two additional index value markers were also selected, systemic inflammation index (SII) and systemic inflammation response index (SIRI) [23, 24]. We then apply the cut off values to a dataset of NSCLC and SCLC patients to determine if the association between high SI values and plasma factors with outcomes. The two types of lung cancer, SCLC and NSCLC, appeared to have disparate immune states and the immune state as related with outcomes for both NSCLC and SCLC groups.

## Materials and methods

### Patients

Patients were consented on a Penn State Cancer Institute Internal Review Board PSI-IRB-approved biomarker study (STUDY00000998). The patients were divided into three groups: subjects who had a surgical resection greater than six month prior to blood collection and no evidence of disease as determined by chest CT; NSCLC, those diagnosed with advanced NSCLC and had received a therapeutic intervention; and SCLC, who received a therapeutic intervention. Blood collection occurred at varying time points for the patients throughout treatment or during follow-up for surgically resected patients with no evidence of disease. Blood cell counts were recorded from the patients chart. SI markers were calculated as follows; neutrophil to lymphocyte ratio (NLR), absolute neutrophil count / absolute lymphocyte count; Platelet to lymphocyte ratio (PLR), absolute platelet count / absolute lymphocyte count; systemic inflammation index (SII), absolute neutrophil count x absolute platelet count / absolute lymphocyte count; systemic inflammatory response index (SIRI), absolute neutrophil count x absolute monocyte count / absolute lymphocyte count.

### Literature search

PubMed was searched on 12/15/2020 using the keywords, "NLR", "Lung Cancer". Articles older than 2017 and those that were meta-analyses were excluded. The top 20 research articles were selected and summarized in S1 Table.

### Multiplex bead-based assay for plasma factor measurements

Plasma factors were quantified using the Milliplex MAP Human Cytokine, Chemokine, and Growth Factor Panel A Magnetic Bead Panel kit (HCYTA-60K, Millipore) according the manufacturers’ protocol. Briefly, the panel included 47 factors measured concurrently. Beads were mix with 12.5 μl of plasma and incubated in a 96 well plate overnight at 4°C with shaking. Bead were collected and washed on a magnet before the plates were read on a MagPix (Millipore) instrument and analyzed using xPONENT software (Millipore). An eight-point standard curve was generated for each plate and two quality control samples were run on each plate to ensure consistency between plates. Each sample was run in duplicate and the average of the two runs was used for the subsequent analysis. Values below the detection limit were set to 0, and the resulting values were log transformed using the equation log_2_(x+1) prior to statistical analysis.

### Analysis and statistics

All analyses and visualizations were performed using the R platform version 3.6.1 [25]. Because SI markers and cytokine factors did not show a normal distribution, based on a Shapiro-Wilk test, the markers were compared using a Kruskal-Wallis test and comparisons between groups was done with a Dunn test.

Kaplan-Meier curves and Cox Proportional Hazards (CPH) models were generated and visualized using the Survival and Survminer packages. Curves were compared using a log-rank test. Forest plots were generated to visualize the results of the CPH models using the ggplot2 package. For all statistical tests, a p-value < 0.05 was considered significant.

## Results

### Systemic markers vary between NSCLC and SCLC patients

In order to gain a better understanding of systemic inflammation (SI) in lung cancer and how those discrimination values are determined, we selected 20 peer-reviewed articles that studied SI markers in lung cancer (summarized in S1 Table). Most of the reports (n = 13) used receiver operator curves (ROC) to determine the optimal SI marker level to differentiate patients, while values from previous studies (n = 5) and median SI marker value (n = 1) were also utilized. Optimized marker values to differentiate SI ranged widely between the different studies, i.e. neutrophil to lymphocyte ratio (NLR) ranged from 1.5–6 (median = 3), this makes establishing clinically useful marker values difficult to standardize.

**Fig 1:**
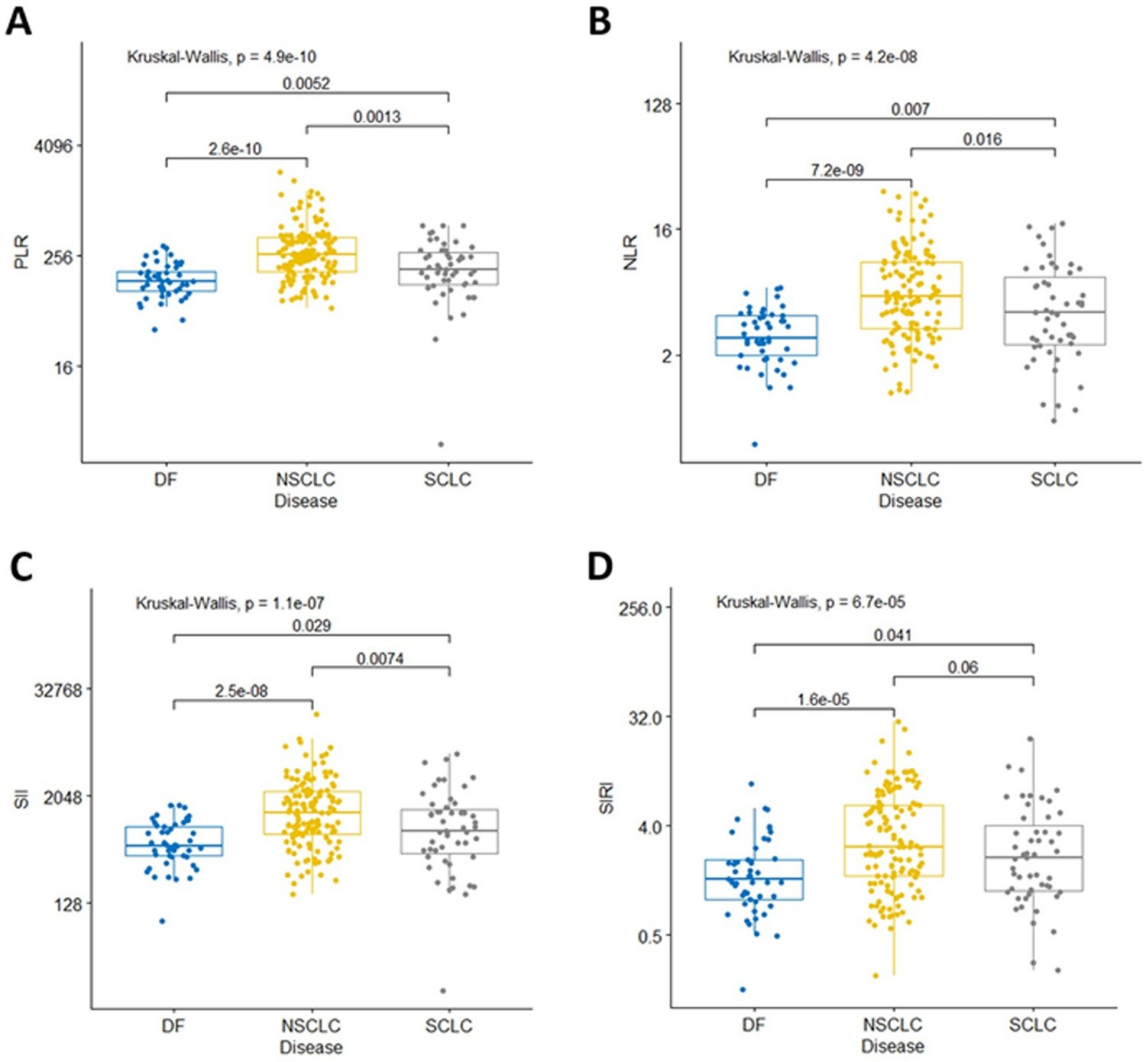
Systemic inflammation markers in our lung cancer cohort. Summary of NLR (A), PLR (B), SII (C) and SIRI (D) values for surgically resected patients with no evidence of disease (DF, n = 49), non-small cell lung cancer (NSCLC, n = 135), and small cell lung cancer (SCLC, n = 51) patients. Groups were compared with a Kruskal-Wallis test and pair-wise comparisons were done with a Dunn test.

We have established a dataset of 235 patients from a clinical study; 51 surgically resected patients with no evidence of disease in the lung (confirmed by CT scan at the time of SI marker measurement), 135 patients with advanced NSCLC, and 49 patients with advanced small cell lung cancer (SCLC), to identify discrete optimized marker levels to differentiate lung cancer-associated SI. Patent characteristics are summarized in S2 Table. SI marker values for the three groups are summarized in S3 Table. NSCLC patients have significantly higher NLR, platelet-to-lymphocyte ratio (PLR), systemic inflammation index (SII), and systemic inflammation response index (SIRI) (p-values are all <0.001) marker values than surgically resected patients with no evidence of disease, and significantly higher NLR, PLR, and SII values (p-values are 0.014, 0.0022, and 0.0089, respectively) than SCLC patients (Fig 1). SCLC patients had significantly higher NLR, PLR, and SII values (p = 0.011, 0.0076, and 0.021, respectively) than surgically resected patients with no evidence of disease (Fig 1). It is interesting to note that SCLC patients seem to have lower levels of SI markers than NSCLC patients, and it seems there is good agreement between SI markers with three of the four markers showing increased SI, relative to subjects without lung cancer, for both lung cancer types. NLR and PLR seem to be the most consistent.

### Establishing and testing discrete SI marker optimized marker value to differentiate SI values

Although SI markers were significantly higher in cancer patients, there was significant overlap between the surgically resected patients with no evidence of disease and the group of active cancer patients (S1 Fig), suggesting that there were two groups of cancer patients; those with low SI levels (similar to surgically resected patients with no evidence of disease) and those with high SI levels (above surgically resected patients). Median NLR and PLR values reported in the literature fell around the middle of our surgically resected patients with no evidence of disease cohort (solid line S1 Fig). Therefore, we established an optimized marker value to differentiate SI based on the 99^th^ percentile of the surgically resected patients with no evidence of disease for each of the SI markers. These values could then be applied to delineate cancer patients with high versus low SI (S3 Table). The NLR and PLR optimized marker values to differentiate SI (6.04 and 320, respectively) were above the maximum values used in the studies found in S1 Table; NLR maximum = 6, PLR maximum = 250. Optimized SI values for all the markers are shown in S3 Table.

**Fig 2:**
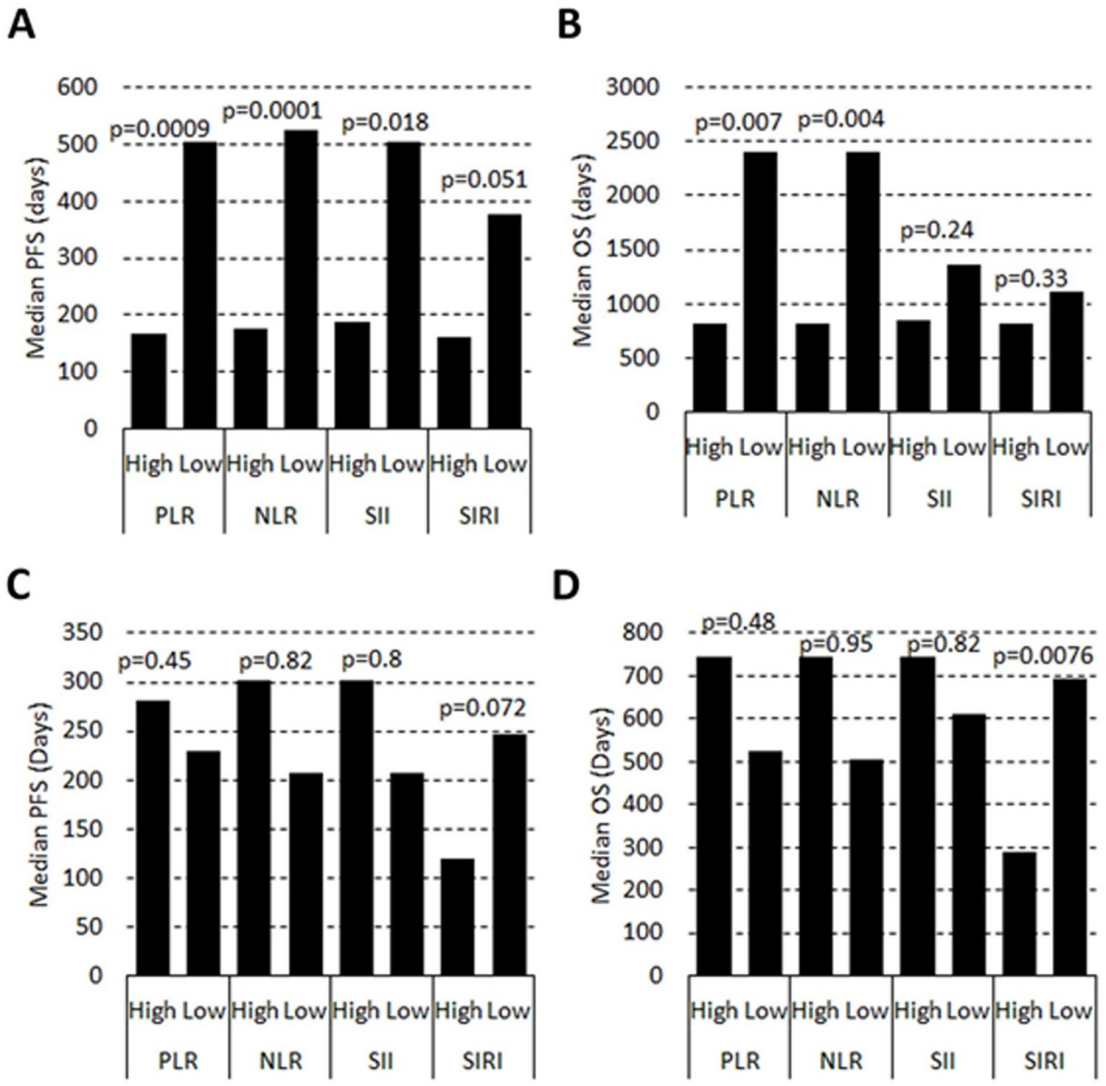
Predicting outcomes in lung cancer using systemic inflammation (SI) markers. NSCLC (A and B) and SCLC (C and D) were separated into high or low SI using the optimized values for each marker based on the 99^th^ percentile of resected patients with no evidence of disease. Progression-free (A and C) and overall (B and D) survival were determined using Kaplan-Meier curves. Median survival is plotted for each marker and the Log-Rank test p-value for each comparison is shown above the bars.

Once the optimized marker value to differentiate SI were established, we sought to determine if lung cancer patients with elevated SI had worse outcomes (progression-free survival (PFS) or overall survival (OS)) than those with lower SI. In NSCLC patients, low levels of NLR, PLR, and SII were associated with a significant increase in PFS (p = <0.001, <0.001, and 0.018, respectively), while SIRI values were not associated with a PFS benefit (Fig 2A). Low NLR and PLR values were associated with an OS benefit, while SII and SIRI were not (Fig 2B). No association between any of the SI markers and PFS was observed in SCLC patients, but low SIRI values were associated with an extended OS (Fig 2C, 2D). However, there were only 4 patients in the SIRI high group.

**Fig 3:**
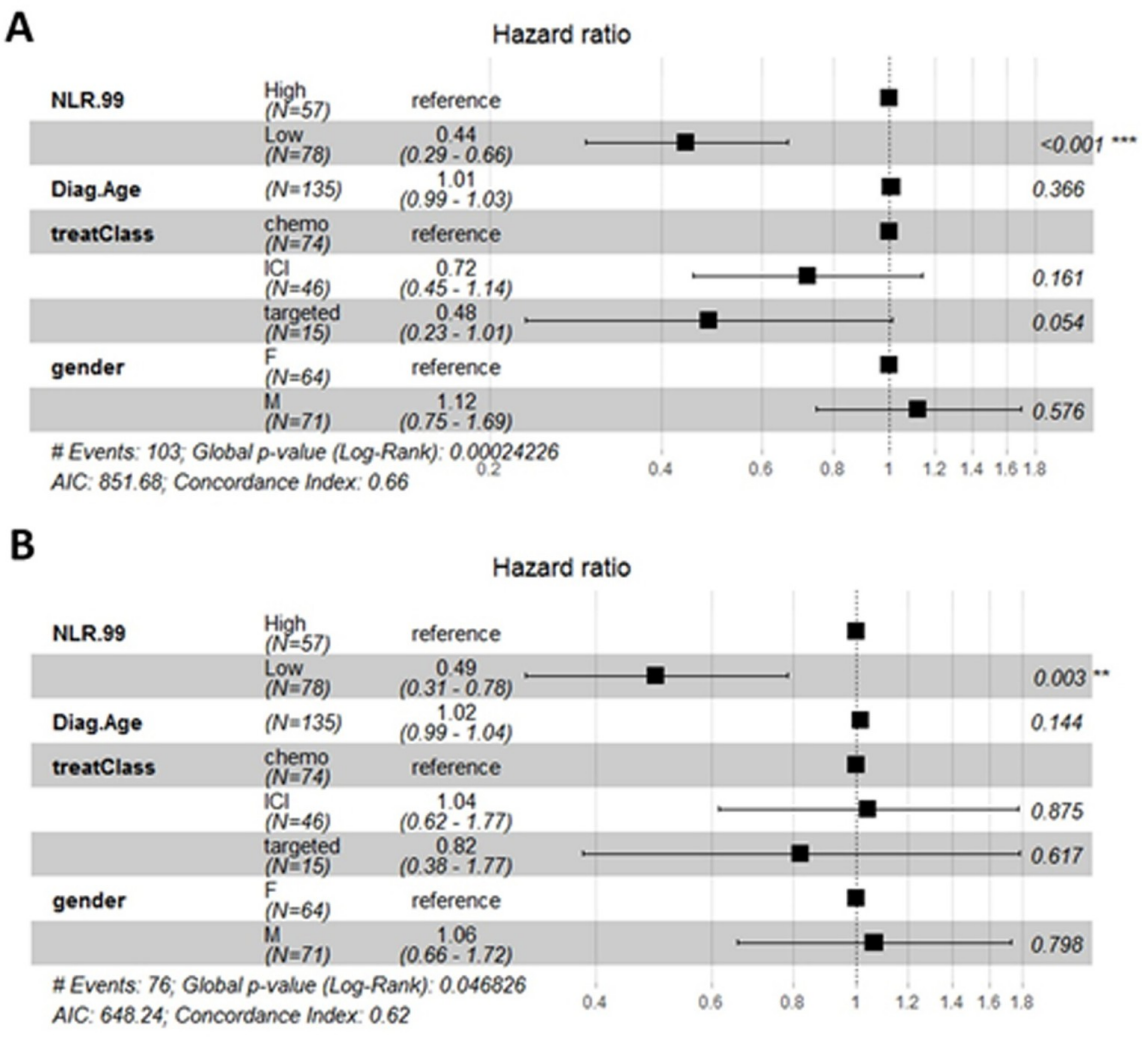
Forest plots to summarize the results of multivariate Cox Proportional Hazards models for NLR. Model results for PFS (A) and OS (B) shown. The model included NLR classification (Low/High), age at diagnosis (Diag.Age), type of treatment (treatClass; chemotherapy (chemo), ICI (immune checkpoint inhibitors), targeted agent (i.e. erlotinib)), and gender. The global model p-value is located under the plot, and p-values for variables are listed to the right.

Our cohort of cancer patients was diverse, so a Cox Proportional Hazards model was applied to the data to determine affects from confounding variables. NLR, PLR, and SII remained the only significant independent predictor of PFS in the model for NSCLC patients (HR = 0.44 (0.29–0.66), p = <0.001; HR = 0.5 (0.33–0.74), p = <0.001; HR = 0.58 (0.39–0.87), p = 0.009, respectively), while age, gender, and treatment type (chemotherapy, immunotherapy, or targeted therapy) were not significantly associated with PFS (Fig 3, S3 Fig). Low SIRI values were found to be significantly associated with an improved PFS (HR = 0.45 (0.27–0.74), p = 0.002) when controlled for compounding variables and treatment with ICIs and targeted were also associated with a better outcome (S2 Fig). Overall survival remained significantly improved for patients with low NLR or PLR when controlled for other variables, while there was no OS benefit for patients with low values of SII and SIRI (Fig 3, S3 Fig). For SCLC patients, gender was the only variable associated with PFS and suggested that the male gender is associated with a shorter PFS (S4 and S5 Figs). Overall, NLR and PLR appear to be the best markers for identifying lung cancer-associated SI, and the values establish and applied in this work to distinguish SI can be useful for predicting PFS and OS in lung cancer patients.

### Circulating cytokines levels and outcomes in NSCLC and SCLC

**Fig 4:**
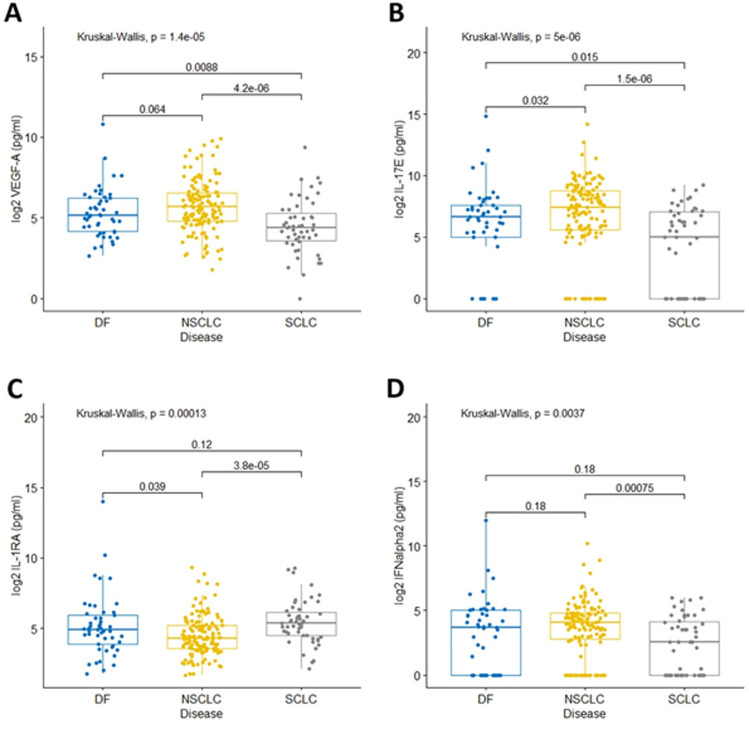
Comparing plasma factor abundances between lung cancer types. Plasma levels of factors that are significantly different between NSCLC and SCLC are plotted, VEGF-A (A), IL-17E (B), IL-1RA (C), and IFNα2 (D). Resected patients with no evidence of disease (DF) are also plotted for comparison. Kruskal-Wallis p-values are displayed at the top of each plot and pair-wise comparisons using a Dunn test are shown.

Since there appeared to be differences in the inflammatory state between NSCLC and SCLC patients based on certain SI markers, we sought to determine if these differences were also reflected in the plasma cytokine profile of these lung cancer patients. Endogenous plasma levels of 47 cytokines, chemokines, and growth factors associated with inflammation and cancer were measured in each patient. We identified four differentially abundant factors in plasma (p<0.01) between NSCLC and SCLC patients, vascular epithelial growth factor-A (VEGF-A, p<0.0001), interleukin 17E (IL-17E, p<0.0001), and interferon alpha2 (IFN-α2, p = 0.0089) were elevated in NSCLC patients and interleukin 1 receptor antagonist (IL-1RA, p = 0.0006) was lower in NSCLC patients (Fig 4). Overall, the resected patients had levels of the four factors that fell between NSCLC and SCLC patients (Fig 4).

All the factors were evaluated for an association with PFS and OS for NSCLC and SCLC patients. Elevated IL-6 was associated with poor PFS and OS, while a weaker association with a poor OS was observed for soluble CD40L (sCD40L), IL-8, and IL-27 in those diagnosed with NSCLC (S5 Table). None of the factors measured in our study were associated with PFS or OS in SCLC patients (S5 Table).

### Immune state heterogeneity in NSCLC patients with poor outcomes

**Fig 5:**
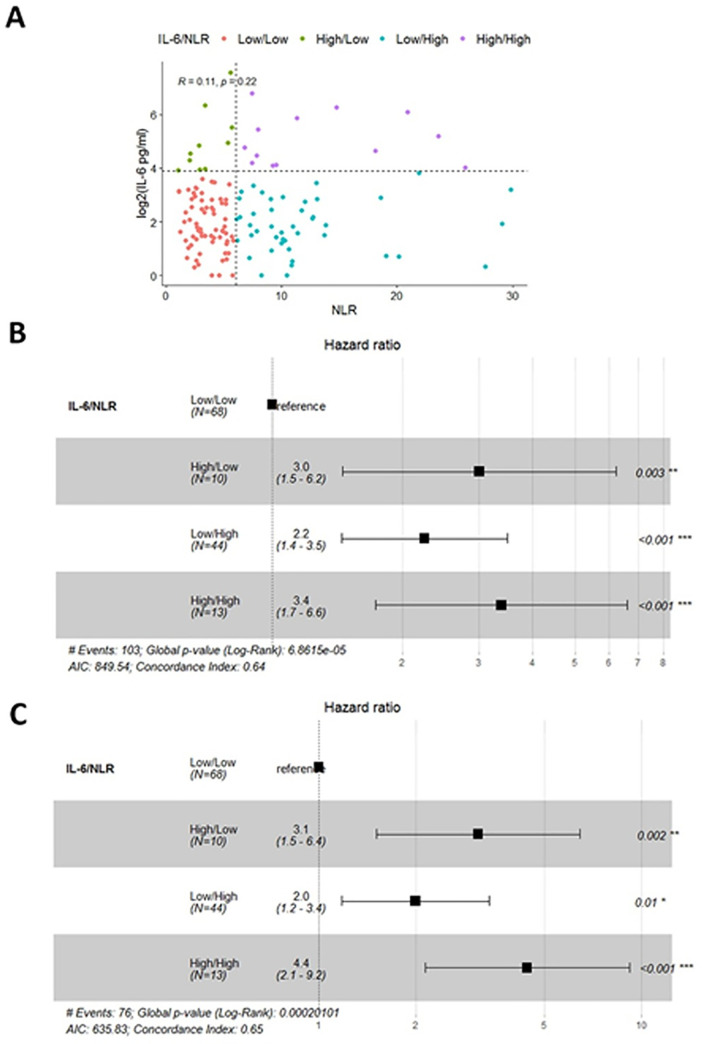
Identifying heterogeneous subsets of NSCLC patients with poor outcomes. A poor correlation between NLR and plasma IL-6 levels was observed in the NSCLC patients (A). Using optimized values to differentiate NLR and IL-6 high and low patients (dashed line) four groups of patients could be identified (A, colored dots). A Cox Proportional Hazards model was used to estimate the hazards of each group for PFS (B) and OS (C).

IL-6 and SI markers (i.e., NLR) both had strong associations with PFS and OS in NSCLC. To determine if these markers are acting together or independently to exert a survival effect, we looked for a correlation between IL-6 and NLR, selected because it was strongest SI marker from our study and the most popular SI marker studied, in NSCLC patients. The correlation between IL-6 and NLR was poor in our cohort (Fig 5A). However, when the data was plotted along with the optimal levels to differentiate patients with Low and High levels of each marker, we observed four distinct groups of NSCLC patients; IL-6^low^/NLR^low^, IL-6^high^/NLR^high^, IL-6^low^/NLR^high^, and IL-6^high^/NLR^low^ (Fig 5A). We performed a Cox Proportional Hazards model analysis to determine if these groups differed in PFS or OS. Elevated levels of IL-6, NLR, or both, resulted in an increased hazard for a shorter progression-free and shorter overall survival relative to the IL-6^low^/NLR^low^ group in NSCLC patients (Fig 5).

**Fig 6:**
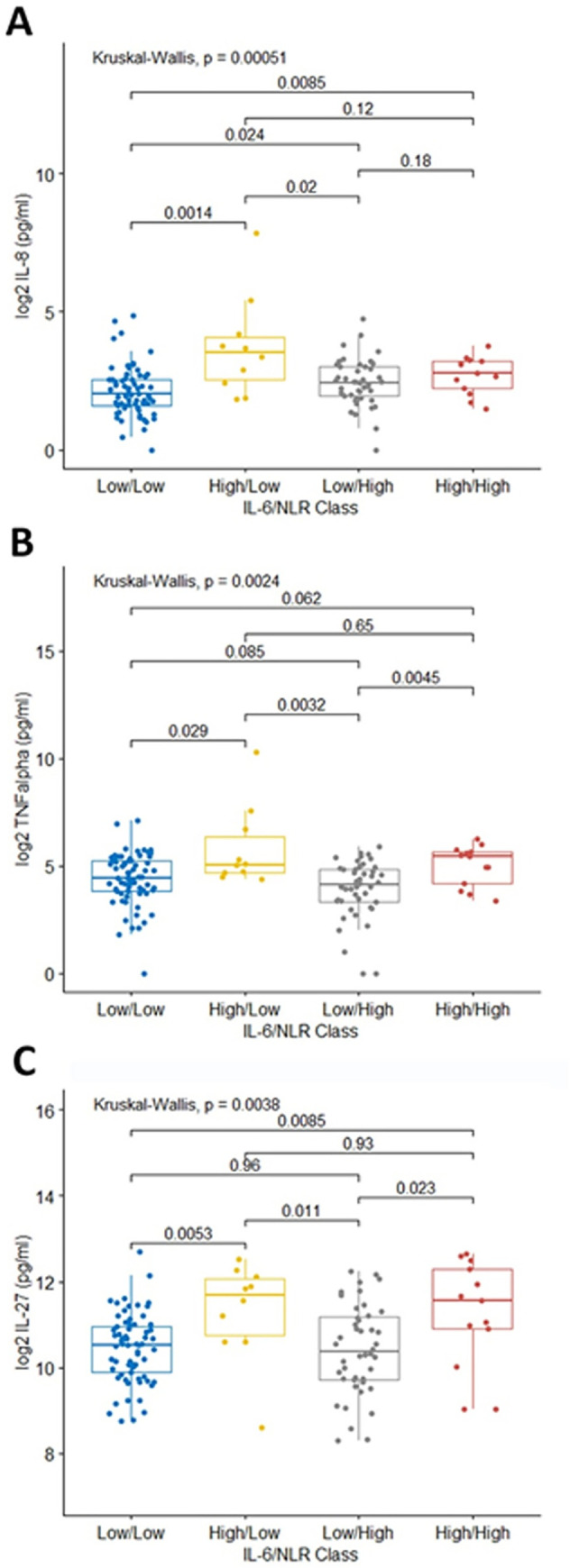
Differential plasma factor abundances between heterogeneous NSCLC subgroups. Three groups of NSCLC patients, high IL-6 (n = 10), high NLR (n = 44), or high IL-6 and high NLR (n = 13), have significantly worse outcomes than those with low IL-6 and NLR (n = 68). The three factors that were found to be significantly different between the groups are plotted, IL-8 (A), TNFα (B), and IL-27 (C). Kruskal-Wallis p-values are displayed at the top of each plot and pair-wise comparisons using a Dunn test are shown.

Next, we explored the plasma cytokine profiles of these IL-6/NLR groups in NSCLC patients to gain insights into differences in immune states between them. Three cytokines, IL-8 (p = 0.012), TNFα (p = 0.038), and IL-27 (p = 0.045), were identified as differentially abundant between the groups (S6 Table). All three of the groups with a short PFS and OS, IL-6^high^/NLR^low^, IL-6^high^/NLR^high^, and IL-6^low^/NLR^high^, had elevated levels of IL-8, relative to the group with the best prognosis, IL-6^low^/NLR^low^ (Fig 6). Groups with high levels of IL-6 tended to have higher levels of TNFα and IL-27 than groups with low IL-6 levels (Fig 6). The three groups with poor outcomes can be defined using the three cytokines relative to the group with the best outcomes (low IL-6^low^/NLR^low^); IL-6^high^/NLR^low^ have elevated IL-8, TNF alpha, and IL-27, IL-6^high^/NLR^low^ has high IL-8 and high IL-27, and IL-6^low^/NLR^high^ group has high IL-8 only.

## Discussion

We explored the immune state of lung cancer patients by measuring four systemic inflammation (SI) markers and 47 plasma cytokines, chemokines, and growth factors in 235 patients to better understand how inflammation effects outcomes in lung cancer. First, we needed to define clinically relevant and broadly applicable optimized SI marker values to differentiate patients with high or low systemic inflammation. Our optimized SI marker value was set at the 99^th^ percentile of patients with resected tumors and no evidence of disease. Values to define SI are typically based on ROC curves derived from the authors’ dataset, but based on our surgically resected patients with no evidence of disease, these values appear to be too low and include a large amount of patients without disease. Therefore, the effect of the SI markers seen in these studies may include artifacts derived from an artificially low SI threshold value. When the new optimized marker values to differentiate SI that are proposed in this study were applied of our NSCLC patients, elevated NLR, PLR, and SII values was associated with a significantly shorter progression-free survival (PFS). None of SI marker were associated with PFS in SCLC patients. While there are some reports that show a relationship between SI markers and PFS or overall survival (OS) in SCLC [10, 12, 17], others fail to establish an association in this type of disease, which may be a result of suboptimal values to define SI [15].

Based on SI markers and plasma factors measured in this study, the immune states between NSCLC and SCLC patients appear to be distinct. SI markers and pro-inflammatory cytokine (IL-17F and IFNα2) tend to be higher in NSCLC patients and the anti-inflammatory cytokine IL-1RA tends to be high in SCLC, which taken together suggests that NSCLC patients tend to have elevated levels of inflammation relative to those diagnosed with SCLC. This observation was surprising based on the fact that SCLC patients tend to have a worse outcome than NSCLC patients, 5 year survival of 6% and 24%, respectively. Neutrophils, a constituent of the SI markers, except PLR, can have an immunosuppressive role in cancer and are associated with angiogenesis and metastasis [26, 27]. Platelets can also contribute to cancer metastasis by secreting factors associated with angiogenesis and tumor growth and by forming microthrombi with circulating tumor cells [27]. IFNα2, like other type I interferons, can inhibit cancer cell proliferation, enhance immune system-mediated cancer cell response, and enhance the efficacy of therapeutic agents [28–31]. IL-17E has been shown to be involved in tumorigenesis and progression, and epithelial/mesenchymal transition (EMT) and increased risk of metastasis [32–34]. It is not clear if or to what extent systemic inflammation and plasma factors directly contribute to the poor prognosis of SCLC patients or the improved outcomes seen in NSCLC.

We confirm previous reports that show SI is a poor prognostic indicator in NSCLC [10, 35, 36]. However, high IL-6 levels are also known to be a poor prognostic indicator in NSCLC, but we did not find a significant direct linear relationship between IL-6 and NLR values in our cohort [20, 21]. Instead, we identified four immune states that existed in our NSCLC patients based on the inflammation marker NLR and IL-6 plasma cytokine levels; Low inflammation and IL-6 (NLR^low^, IL-6^low^), low inflammation and high IL-6 (NLR^low^, IL-6^high^), high inflammation and low IL-6 (NLR^high^, IL-6^low^), and high inflammation and high IL-6 (NLR^high^, IL-6^high^). Patients with low IL-6 and NLR values had a better prognosis than those with high levels of either IL-6 or NLR or both IL-6 and NLR. These subgroups of patients with a poor prognosis, NLR^low^/ IL-6^high^, NLR^high^/ IL-6^low^, and NLR^high^, IL-6^high^, can be distinguished based on plasma levels of IL-8, TNFα, and IL-27. Each of these cytokine are associated with detrimental effects on the immune system in the tumor microenvironment. IL-8 can attract immune cells to the tumor microenvironment and suppress immune responses, can facilitate EMT, and has been shown to limit the efficacy of immune checkpoint inhibitors (ICIs) [37, 38]. High levels of TNFα are associated with immune system escape and tumor progression or the development of ICI resistance [39, 40]. IL-27 can provide anti-tumor effects but also can down-regulate T cells and up-regulate immune checkpoint proteins PD-1 and PD-L1 [41]. While these plasma factors are associated with a worse prognosis in NSCLC patients, we cannot conclude the extent to which they are directly affecting survival in these subgroups.

The discovery of these subgroups of patients with a poor prognosis could have significant treatment implications. The IL-6^high^/NLR^low^ group of patients may benefit from combining a standard of care therapeutic with an IL-6 inhibitor. NLR^high^/IL-6^low^ group of patients may benefit from a standard treatment with corticosteroids to reduce systemic inflammation, and NLR^high^/IL-6^high^ group of patients may achieve improved outcomes from combining standard therapy with an IL-6 inhibitor and a corticosteroid. These form the rationale for developing precision therapies for patients with lung cancer.

Here we compared the systemic immune status of NSCLC and SCLC patients and established optimized SI marker values for NLR, PLR, SII, and SIRI to distinguish those with high or low SI. SCLC patients tended to have lower levels of inflammation than those with NSCLC, and systemic inflammation is associated with worse outcomes in NSCLC but not SCLC. We also identified heterogeneity within the NSCLC patients with a poor prognoses based on systemic inflammation markers and IL-6 levels. This heterogeneity could be exploited to personalize treatment based on a patients’ immune state by including IL-6 inhibitors or steroids.

## Supporting information

S1 Fig Comparison of SI markers in different groups of lung cancer patients. NLR and PLR were plotted for disease-free (DF), non-small cell lung cancer (NSCLC) and small cell lung cancer (SCLC) patients (A). Median cutoff values for NLR and PLR from the literature are shown with the solid black line (3 and 148.6, respectively), and new cutoff values based on the 99^th^ percentile of disease-free patients (6.04 and 320, respectively) are shown with the dashed line. Patients with high levels of SI markers based on the new cutoff values are shown in blue. SII and SIRI were plotted for the same patient groups as A (B). The 99^th^ percentile of disease-free patients for SII (1615) and SIRI (7.3) are marked with the dashed line.
(TIF)

S2 Fig Forest plots to summarize the results of multivariate Cox Proportional Hazards models for various SI markers for PFS in NSCLC. Models for PLR (A), SII (B), and SIRI (C) are shown. The model included SI classification (Low/High), age at diagnosis (Diag.Age), type of treatment (treatClass; chemotherapy (chemo), ICI (immune checkpoint inhibitors), targeted agent (i.e. erlotinib)), and gender.
(TIF)

S3 Fig Forest plots to summarize the results of multivariate Cox Proportional Hazards models for various SI markers for OS in NSCLC. Models for PLR (A), SII (B), and SIRI (C) are shown. The model included SI classification (Low/High), age at diagnosis (Diag.Age), type of treatment (treatClass; chemotherapy (chemo), ICI (immune checkpoint inhibitors), targeted agent (i.e. erlotinib)), and gender.
(TIF)

S4 Fig Forest plots to summarize the results of multivariate Cox Proportional Hazards models for various SI markers for PFS in SCLC. Models for NLR (A), PLR (B), SII (C), and SIRI (D) are shown. The model included SI classification (Low/High), age at diagnosis (Diag.Age), type of treatment (treatClass; chemotherapy (chemo), ICI (immune checkpoint inhibitors), targeted agent (i.e. erlotinib)), and gender.
(TIF)

S5 Fig Forest plots to summarize the results of multivariate Cox Proportional Hazards models for various SI markers for OS in SCLC. Models for NLR (A), PLR (B), SII (C), and SIRI (D) are shown. The model included SI classification (Low/High), age at diagnosis (Diag.Age), type of treatment (treatClass; chemotherapy (chemo), ICI (immune checkpoint inhibitors), targeted agent (i.e. erlotinib)), and gender.
(TIF)

S1 Table Summary of selected articles evaluating systemic inflammation (SI) markers in lung cancer. (PDF)

S2 Table Summary of patient characteristics who were included in the study. (PDF)

S3 Table Summary of SI markers and classification in the different patient groups. (PDF)

S4 Table Median progression-free survival for SI markers based on Kaplan-Meier analysis. (PDF)

S5 Table Progression-free (PFS) and overall (OS) survival effects of individual cytokines for NSCLC and SCLC with p-values adjusted using the Benjamini-Hochberg method. (PDF)

S6 Table Kruskal-Wallis test results comparing each plasma factor between the four IL-6/NLR groups with multi-sample adjustment using Benjamini-Hochberg method. (PDF)

## Abbreviations

SI: systemic inflammation
NLR: neutrophil to lymphocyte ratio
PLR: platelet to lymphocyte ratio
SII: systemic inflammation index
SIRI: systemic inflammatory response index
OS: overall survival
PFS: progression-free survival
ICI: immune checkpoint inhibitors
