# Supplementary figures and images for: Diversity and heterogeneity of immune states in non-small cell lung cancer and small cell lung cancer
Source: PLoS One. 2021 Dec 2;16(12):e0260988. doi: 10.1371/journal.pone.0260988 (PMC8638918; doi:10.1371/journal.pone.0260988)

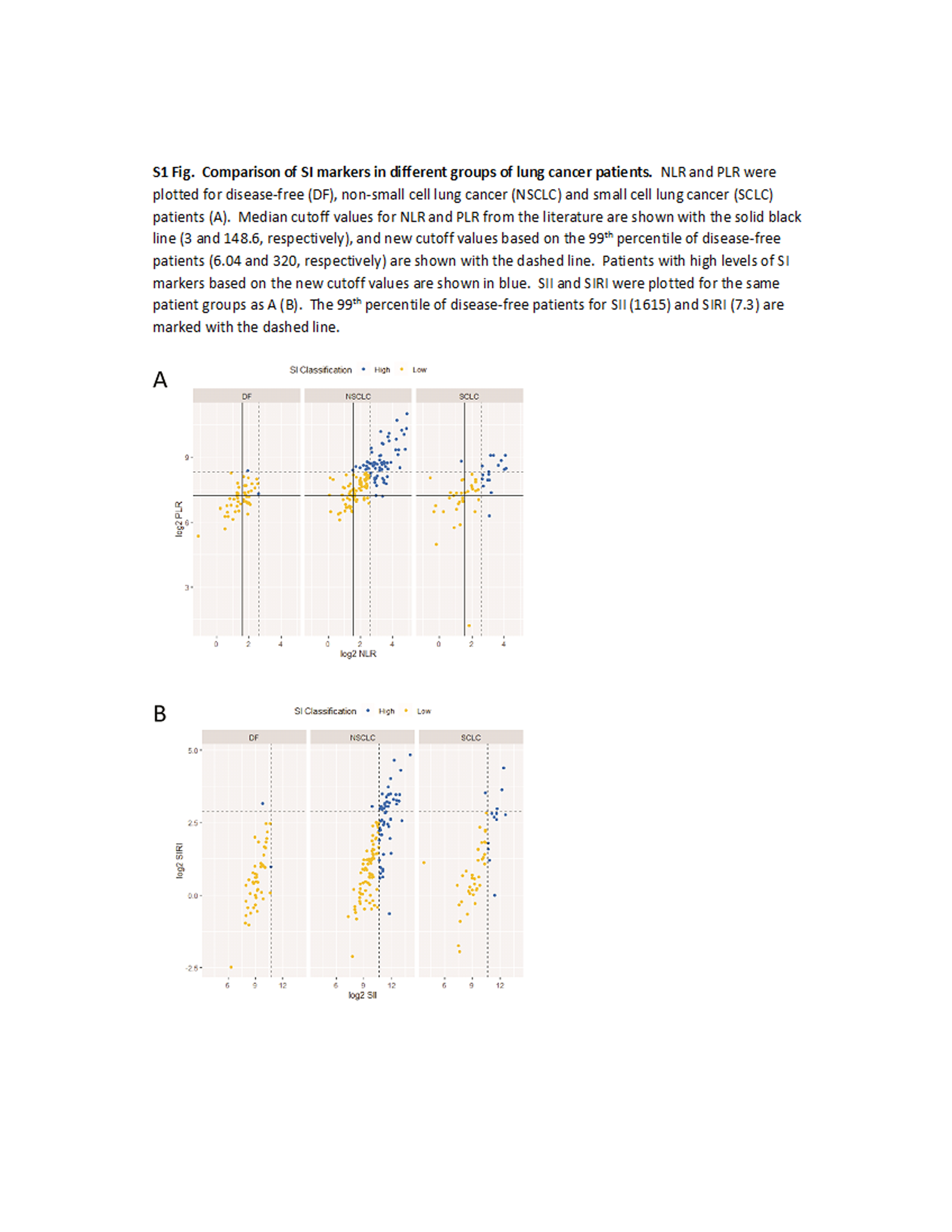

Supplement: S1 Fig — NLR and PLR were plotted for disease-free (DF), non-small cell lung cancer (NSCLC) and small cell lung cancer (SCLC) patients (A). Median cutoff values for NLR and PLR from the literature are shown with the solid black line (3 and 148.6, respectively), and new cutoff values based on the 99th percentile of disease-free patients (6.04 and 320, respectively) are shown with the dashed line. Patients with high levels of SI markers based on the new cutoff values are shown in blue. SII and SIRI were plotted for the same patient groups as A (B). The 99th percentile of disease-free patients for SII (1615) and SIRI (7.3) are marked with the dashed line. (TIF) [file pone.0260988.s001.tif]

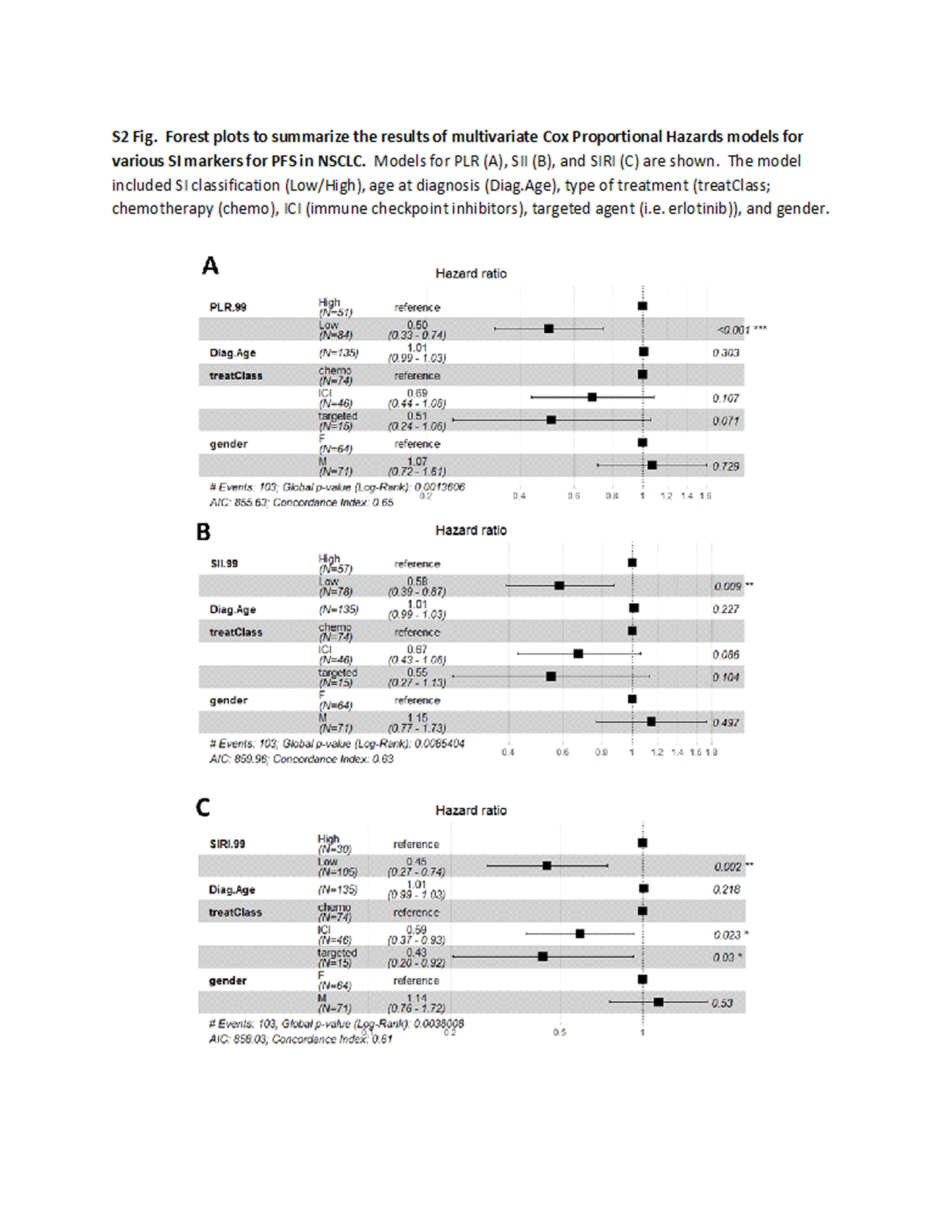

Supplement: S2 Fig — Models for PLR (A), SII (B), and SIRI (C) are shown. The model included SI classification (Low/High), age at diagnosis (Diag.Age), type of treatment (treatClass; chemotherapy (chemo), ICI (immune checkpoint inhibitors), targeted agent (i.e. erlotinib)), and gender. (TIF) [file pone.0260988.s002.tif]

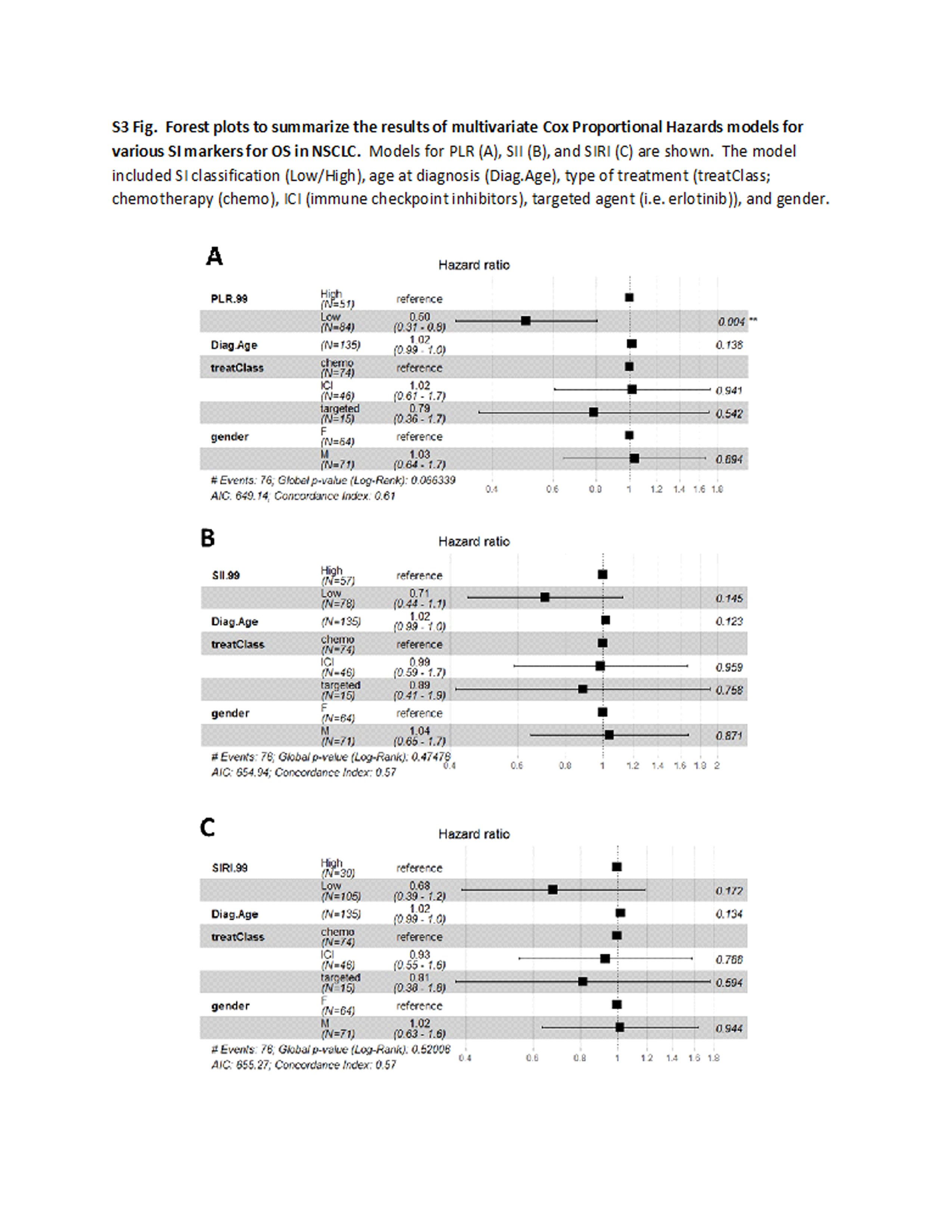

Supplement: S3 Fig — Models for PLR (A), SII (B), and SIRI (C) are shown. The model included SI classification (Low/High), age at diagnosis (Diag.Age), type of treatment (treatClass; chemotherapy (chemo), ICI (immune checkpoint inhibitors), targeted agent (i.e. erlotinib)), and gender. (TIF) [file pone.0260988.s003.tif]

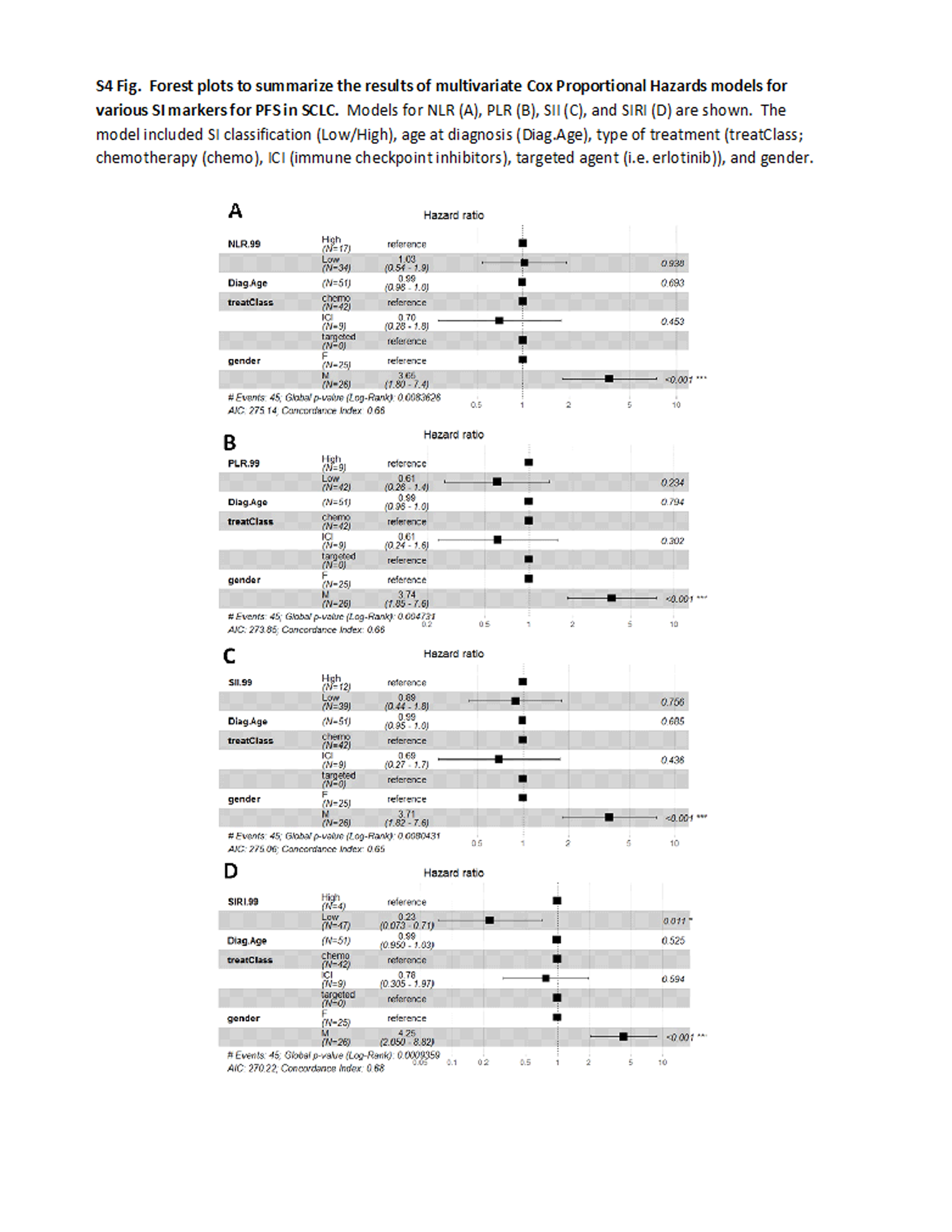

Supplement: S4 Fig — Models for NLR (A), PLR (B), SII (C), and SIRI (D) are shown. The model included SI classification (Low/High), age at diagnosis (Diag.Age), type of treatment (treatClass; chemotherapy (chemo), ICI (immune checkpoint inhibitors), targeted agent (i.e. erlotinib)), and gender. (TIF) [file pone.0260988.s004.tif]

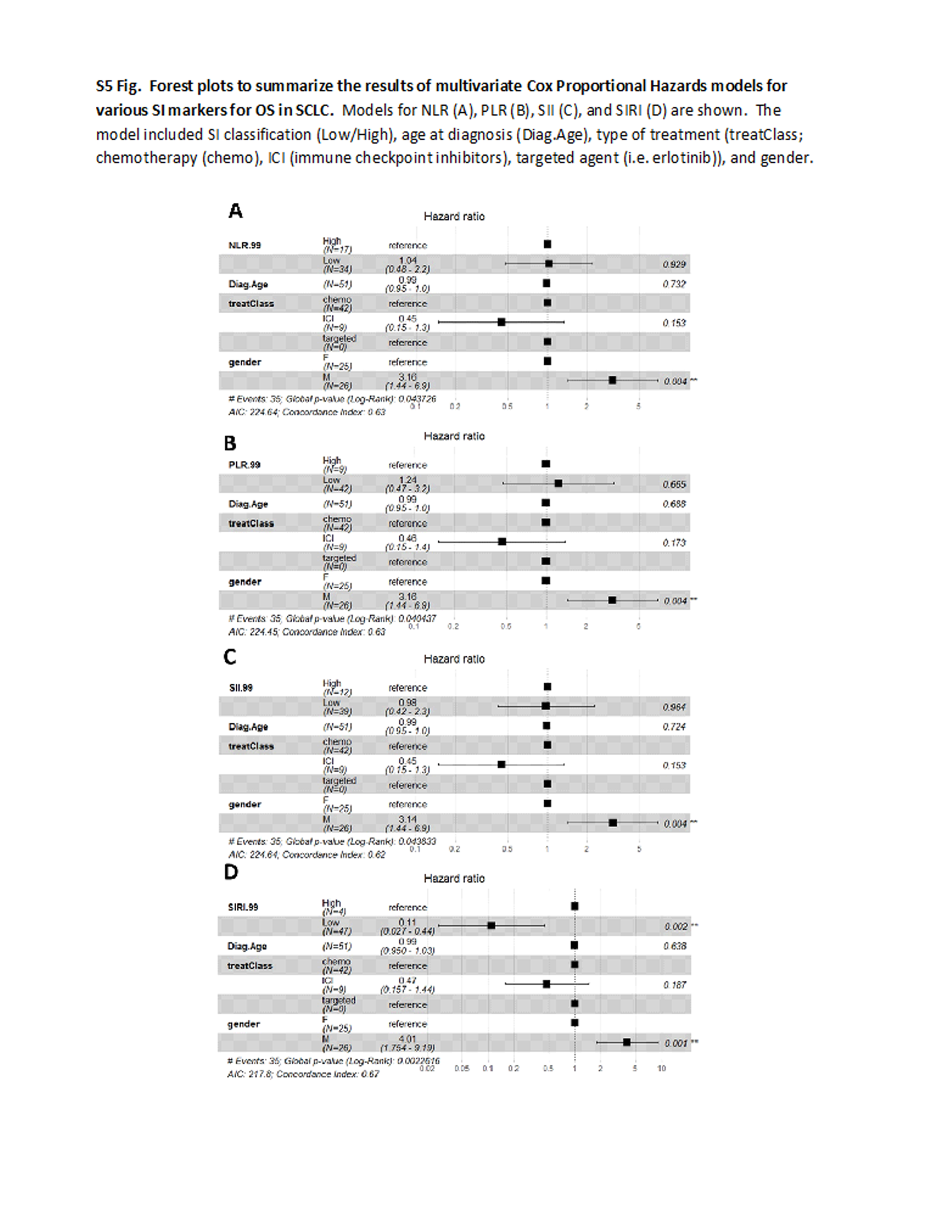

Supplement: S5 Fig — Models for NLR (A), PLR (B), SII (C), and SIRI (D) are shown. The model included SI classification (Low/High), age at diagnosis (Diag.Age), type of treatment (treatClass; chemotherapy (chemo), ICI (immune checkpoint inhibitors), targeted agent (i.e. erlotinib)), and gender. (TIF) [file pone.0260988.s005.tif]
