# Supplementary material for: Diversity and heterogeneity of immune states in non-small cell lung cancer and small cell lung cancer
Source: PLoS One. 2021 Dec 2;16(12):e0260988. doi: 10.1371/journal.pone.0260988 (PMC8638918; doi:10.1371/journal.pone.0260988)
Supplement: S1 Table — (PDF) [file pone.0260988.s006.pdf]

Supplemental Tables

S1 Table. Summary of selected articles evaluating Systemic Inflammation (SI) markers in lung cancer.

Author	Date	Study Design	Patients	Cohort	SI Marker(s)	Cut-off	Cut-off methods	Outcome
Derman et al.(1)	2017	Retrospective	139	Chemo-naïve NSCLC	NLR	5	Previous studies	OS
Zhang et al.(2)	2018	Retrospective	127	EGFR+ NSCLC	NLR, LMR	2.9, 3.37	ROC curve	PFS, OS
Gao et al.(3)	2017	Retrospective	358	NSCLC	NLR, PLR, LMR	2.14, 121.78, 3.06	ROC curve	OS
Maymani et al.(4)	2018	Retrospective	74	NSCLC, IO-treated	NLR	6	Not reported	PFS, OS
Ren et al.(5)	2019	Retrospective	147	NSCLC, IO-treated	NLR	2.5	ROC curve	PFS, OS
Ozyurek et al.(6)	2017	Retrospective	386	NSCLC, SCLC	NLR	3	Previous studies	OS
Wang et al.(7)	2017	Retrospective	695	NSCLC, SCLC	NLR, PLR	6, 248	ROC curve	OS
Tong et al.(8)	2017	Retrospective	332	NSCLC, Stage III	NLR, PLR, SII	3.57, 147, 660	ROC curve	OS
Nakaya et al.(9)	2018	Retrospective	101	NSCLC, nivolumab-treated	NLR	3	Previous studies	PFS
Takeda et al.(10)	2018	Retrospective	30	NSCLC, nivolumab-treated	NLR, PLR	5, 150	Previous studies	PFS
Liu et al.(11)	2019	Retrospective	44	NSCLC, nivolumab-treated	SII, NLR, PLR	603.5, 3.07, 144	ROC curve	PFS, OS
Lan et al.(12)	2017	Retrospective	174	Resected NSCLC	NLR, PLR	2.9, 148.6	ROC curve	PC, OS
Yuan et al.(13)	2017	Retrospective	1466	Resected NSCLC	NLR, PLR, MLR	2.06, 204, 0.35	ROC curve	OS
Huang et al.(14)	2018	Retrospective	589	Resected NSCLC	NLR	2.3	ROC curve	DFS, OS
Wang et al.(15)	2019	Retrospective	235	Resected NSCLC	NLR	2.3	Median	DFS, OS
Zhu et al.(16)	2019	Retrospective	101	Respectable lung cancer	NLR, PLR	2.049, 133.534	ROC curve	OS
Mizuguchi et al.(17)	2018	Retrospective	382	Respectable lung cancer	NLR	1.5, 3.5	ROC curve	OS
Liu et al.(18)	2017	Retrospective	139	SCLC	NLR, PLR	4.55, 148	ROC curve	OS
Drpa et al.(19)	2020	Retrospective	140	SCLC	NLR, PLR, LMR	4 & 5,150 & 250, 2.64 & 4.19	Previous studies	PFS, OS
Shi et al.(20)	2020	Retrospective	106	LCNEC	NLR, PLR	2.52, 133.56	ROC curve	OS

ROC, Receiver operating characteristic; PC, pulmonary complications; LIPI, Lung Immune Prognostic Index; SCLC, small cell lung cancer; NSCLC, non-small cell lung cancer; LCNEC, large cell neuroendocrine carcinoma; SI, systemic inflammation; OS, overall survival; PFS, progression-free survival; NLR, neutrophil to lymphocyte ratio; PLR, platelet to lymphocyte ratio; LMR, lymphocyte to monocyte ratio; MLR, monocyte to lymphocyte ratio.

References

1. Derman BA, Macklis JN, Azeem MS, Sayidine S, Basu S, Batus M*, et al.* Relationships between longitudinal neutrophil to lymphocyte ratios, body weight changes, and overall survival in patients with non-small cell lung cancer. BMC Cancer **2017**;17(1):141 doi 10.1186/s12885-017-3122-y.
2. Zhang Y, Feng YC, Zhu HG, Xiong TC, Hou YS, Song J*, et al.* The peripheral blood neutrophil-to-lymphocyte ratio is a prognostic predictor for survival of EGFR-mutant nonsmall cell lung cancer patients treated with EGFR-TKIs. Medicine (Baltimore) **2018**;97(30):e11648 doi 10.1097/MD.0000000000011648.
3. Gao Y, Zhang H, Li Y, Wang D, Ma Y, Chen Q. Preoperative pulmonary function correlates with systemic inflammatory response and prognosis in patients with non-small cell lung cancer: results of a single-institution retrospective study. Oncotarget **2017**;8(16):27489-501 doi 10.18632/oncotarget.14225.
4. Maymani H, Hess K, Groisberg R, Hong DS, Naing A, Piha-Paul S*, et al.* Predicting outcomes in patients with advanced non-small cell lung cancer enrolled in early phase immunotherapy trials. Lung Cancer **2018**;120:137-41 doi 10.1016/j.lungcan.2018.03.020.
5. Ren F, Zhao T, Liu B, Pan L. Neutrophil-lymphocyte ratio (NLR) predicted prognosis for advanced non-small-cell lung cancer (NSCLC) patients who received immune checkpoint blockade (ICB). Onco Targets Ther **2019**;12:4235-44 doi 10.2147/OTT.S199176.
6. Ozyurek B, Ozdemirel T, Ozden S, Erdogan Y, Kaplan B, Kaplan T. Prognostic Value of the Neutrophil to Lymphocyte Ratio (NLR) in Lung Cancer Cases. Asian Pac J Cancer Prev **2017**;18(5):1417-21 doi 10.22034/APJCP.2017.18.5.1417.
7. Wang L, Liang D, Xu X, Jin J, Li S, Tian G*, et al.* The prognostic value of neutrophil to lymphocyte and platelet to lymphocyte ratios for patients with lung cancer. Oncol Lett **2017**;14(6):6449-56 doi 10.3892/ol.2017.7047.
8. Tong YS, Tan J, Zhou XL, Song YQ, Song YJ. Systemic immune-inflammation index predicting chemoradiation resistance and poor outcome in patients with stage III non-small cell lung cancer. J Transl Med **2017**;15(1):221 doi 10.1186/s12967-017-1326-1.
9. Nakaya A, Kurata T, Yoshioka H, Takeyasu Y, Niki M, Kibata K*, et al.* Neutrophil-to-lymphocyte ratio as an early marker of outcomes in patients with advanced non-small-cell lung cancer treated with nivolumab. Int J Clin Oncol **2018**;23(4):634-40 doi 10.1007/s10147-018-1250-2.
10. Takeda T, Takeuchi M, Saitoh M, Takeda S. Neutrophil-to-lymphocyte ratio after four weeks of nivolumab administration as a predictive marker in patients with pretreated non-small-cell lung cancer. Thorac Cancer **2018**;9(10):1291-9 doi 10.1111/1759-7714.12838.
11. Liu J, Li S, Zhang S, Liu Y, Ma L, Zhu J*, et al.* Systemic immune-inflammation index, neutrophil-to-lymphocyte ratio, platelet-to-lymphocyte ratio can predict clinical outcomes in patients with metastatic non-small-cell lung cancer treated with nivolumab. J Clin Lab Anal **2019**;33(8):e22964 doi 10.1002/jcla.22964.
12. Lan H, Zhou L, Chi D, Zhou Q, Tang X, Zhu D*, et al.* Preoperative platelet to lymphocyte and neutrophil to lymphocyte ratios are independent prognostic factors for patients undergoing lung cancer radical surgery: A single institutional cohort study. Oncotarget **2017**;8(21):35301-10 doi 10.18632/oncotarget.13312.
13. Yuan C, Li N, Mao X, Liu Z, Ou W, Wang SY. Elevated pretreatment neutrophil/white blood cell ratio and monocyte/lymphocyte ratio predict poor survival in patients with curatively resected non-small cell lung cancer: Results from a large cohort. Thorac Cancer **2017**;8(4):350-8 doi 10.1111/1759-7714.12454.

14. Huang W, Wang S, Zhang H, Zhang B, Wang C. Prognostic significance of combined fibrinogen concentration and neutrophil-to-lymphocyte ratio in patients with resectable non-small cell lung cancer. Cancer Biol Med **2018**;15(1):88-96 doi 10.20892/j.issn.2095-3941.2017.0124.
15. Wang X, Cao L, Li S, Wang F, Huang D, Jiang R. Combination of PD-L1 expression and NLR as prognostic marker in patients with surgically resected non-small cell lung cancer. J Cancer **2019**;10(26):6703-10 doi 10.7150/jca.34469.
16. Zhu J, Lian L, Qin H, Wang WJ, Ren R, Xu MD*, et al.* Prognostic evaluation of patients with resectable lung cancer using systemic inflammatory response parameters. Oncol Lett **2019**;17(2):2244-56 doi 10.3892/ol.2018.9858.
17. Mizuguchi S, Izumi N, Tsukioka T, Komatsu H, Nishiyama N. Neutrophil-lymphocyte ratio predicts recurrence in patients with resected stage 1 non-small cell lung cancer. J Cardiothorac Surg **2018**;13(1):78 doi 10.1186/s13019-018-0763-0.
18. Liu D, Huang Y, Li L, Song J, Zhang L, Li W. High neutrophil-to-lymphocyte ratios confer poor prognoses in patients with small cell lung cancer. BMC Cancer **2017**;17(1):882 doi 10.1186/s12885-017-3893-1.
19. Drpa G, Sutic M, Baranasic J, Jakopovic M, Samarzija M, Kukulj S*, et al.* Neutrophil-to-lymphocyte ratio can predict outcome in extensive-stage small cell lung cancer. Radiol Oncol **2020**;54(4):437-46 doi 10.2478/raon-2020-0054.
20. Shi M, Zhao W, Zhou F, Chen H, Tang L, Su B*, et al.* Neutrophil or platelet-to-lymphocyte ratios in blood are associated with poor prognosis of pulmonary large cell neuroendocrine carcinoma. Transl Lung Cancer Res **2020**;9(1):45-54 doi 10.21037/tlcr.2020.01.17.
