# Supplementary material for: Diversity and heterogeneity of immune states in non-small cell lung cancer and small cell lung cancer
Source: PLoS One. 2021 Dec 2;16(12):e0260988. doi: 10.1371/journal.pone.0260988 (PMC8638918; doi:10.1371/journal.pone.0260988)
Supplement: S2 Table — (PDF) [file pone.0260988.s007.pdf]

S2 Table. Summary of patient characteristics who were included in the study.

	Surgically resected patients with no evidence of disease (n = 49)	NSCLC (n = 135)	SCLC (n = 51)
**Gender**			
Female	28 (57.1)	64 (47.4)	25 (49.0)
Male	21 (42.9)	71 (52.6)	26 (51.0)
**Age at Diagnosis**			
Median (IQR)	67 (59.00, 72.00)	65 (59.00, 71.00)	66 (59.50, 71.00)
Mean (SD)	65.7 (10.6)	64.6 (10.8)	64.6 (9.0)
**Histology**			
AC	27 (55.1)	88 (65.2)	NA
AC/SC	2 (4.1)	0 (0.0)	NA
BAC	1 (2.0)	0 (0.0)	NA
NEC	3 (6.1)	0 (0.0)	NA
NOS	3 (6.1)	5 (3.7)	NA
SC	13 (26.5)	41 (30.4)	NA
SCLC	0 (0.0)	NA	51 (100.0)
**Time from Start of treatment to blood collection**			
Median (IQR)	NA	35 (8.00, 137.50)	61 (21.00, 101.50)
Mean (SD)	NA	183.71 (364.07)	81.18 (100.99)
**Treatment**			
Chemotherapy	NA	74 (54.8)	42 (82.4)
ICI	NA	46 (34.1)	9 (17.6)
Targeted	NA	15 (11.1)	0 (0.0)
**Line of Treatment**			
1	NA	112 (83.0)	43 (84.3)
2	NA	18 (13.3)	8 (15.7)
>2	NA	5 (3.7)	0 (0.0)

NSCLC, non-small cell lung cancer; SCLC, small cell lung cancer; IQR, interquartile range; SD, standard deviation; AC, adenocarcinoma; AC/SC, adeno-squamous carcinoma; BAC, Bronchioloalveolar carcinoma; NEC, neuroendocrine carcinoma; NOS, non-small cell lung cancer not otherwise specified; SC, squamous cell carcinoma; ICI, immune checkpoint inhibitors (with or without chemotherapy); NA, not applicable.
