# Supplementary material for: Diversity and heterogeneity of immune states in non-small cell lung cancer and small cell lung cancer
Source: PLoS One. 2021 Dec 2;16(12):e0260988. doi: 10.1371/journal.pone.0260988 (PMC8638918; doi:10.1371/journal.pone.0260988)
Supplement: S3 Table — (PDF) [file pone.0260988.s008.pdf]

**S3 Table. Summary of SI markers and classification in the different patient groups.**

| | Disease-free (n = 49) | NSCLC (n = 135) | SCLC (n = 51) |
|---|---|---|---|
| **NLR** | | | |
| Min. | 0.46 | 1.07 | 0.67 |
| Median (IQR) | 2.84 (2.12, 3.80) | 5.24 (3.07, 9.36) | 4.01 (2.38, 7.22) |
| Mean (SD) | 3.01 (1.26) | 7.18 (5.91) | 5.28 (4.25) |
| Max. | 6.11 | 29.83 | 17.51 |
| $99^{th}$ Percentile | 6.04 | NA | NA |
| **NLR Classification** | | | |
| High (n(%)) | NA | 57 (42.2) | 17 (33.3) |
| Low (n(%)) | NA | 78 (57.8) | 34 (66.7) |
| **PLR** | | | |
| Min. | 40.55 | 68.97 | 2.3 |
| Median (IQR) | 135.45 (107.09, 172.36) | 260.00 (172.39, 401.40) | 180.72 (125.80, 276.02) |
| Mean (SD) | 149.09 (64.37) | 349.16 (306.73) | 217.11 (132.62) |
| Max. | 329.77 | 2,083.33 | 548.21 |
| $99^{th}$ Percentile | 320 | NA | NA |
| **PLR Classification** | | | |
| High (n(%)) | NA | 51 (37.8) | 9 (17.6) |
| Low (n(%)) | NA | 84 (62.2) | 42 (82.4) |
| **SII** | | | |
| Min. | 81.5 | 159.75 | 13.42 |
| Median (IQR) | 554.27 (442.28, 926.38) | 1,309.91 (759.47, 2,278.69) | 821.79 (463.30, 1,437.62) |
| Mean (SD) | 685.20 (370.39) | 1,908.62 (2,089.45) | 1,302.05 (1,333.21) |
| Max. | 1,629.07 | 16,858.67 | 6,126.80 |
| $99^{th}$ Percentile | 1615 | NA | NA |
| **SII Classification** | | | |
| High (n(%)) | NA | 57 (42.2) | 12 (23.5) |
| Low (n(%)) | NA | 78 (57.8) | 39 (76.5) |
| **SIRI** | | | |
| Min. | 0.18 | 0.23 | 0.26 |
| Median (IQR) | 1.52 (0.99, 2.08) | 2.68 (1.53, 5.95) | 2.16 (1.15, 4.08) |
| Mean (SD) | 1.97 (1.61) | 4.51 (4.53) | 3.40 (3.71) |
| Max. | 8.96 | 28.78 | 20.87 |
| $99^{th}$ Percentile | 7.3 | NA | NA |
| **SIRI Classification** | | | |
| High (n(%)) | NA | 30 (22.2) | 4 (7.8) |
| Low (n(%)) | NA | 105 (77.8) | 47 (92.2) |
