# Supplementary material for: Diversity and heterogeneity of immune states in non-small cell lung cancer and small cell lung cancer
Source: PLoS One. 2021 Dec 2;16(12):e0260988. doi: 10.1371/journal.pone.0260988 (PMC8638918; doi:10.1371/journal.pone.0260988)
Supplement: S4 Table — (PDF) [file pone.0260988.s009.pdf]

S4 Table. Median Progression-free survival for SI markers based on Kaplan-Meier analysis.

Marker(s)	SI Class	n	Events	Median PFS	0.95 LCL	0.95 UCL	p-value*
NLR	High	57	48	177	117	308	0.0001
	Low	78	55	525	378	748	
PLR	High	51	43	168	112	332	0.0009
	Low	84	60	505	314	731	
SII	High	57	45	189	127	332	0.018
	Low	78	58	505	363	713	
SIRI	High	30	23	161	105	702	0.051
	Low	105	80	378	251	616	

* p-value from a log-rank test; LCL, lower confidence limit UCL, upper confidence limit

Table 5. Progression-free (PFS) and overall (OS) survival effects of individual cytokines for NSCLC and SCLC with p-values adjusted using the Benjamini-Hochberg method.
