# Supplementary material for: Diversity and heterogeneity of immune states in non-small cell lung cancer and small cell lung cancer
Source: PLoS One. 2021 Dec 2;16(12):e0260988. doi: 10.1371/journal.pone.0260988 (PMC8638918; doi:10.1371/journal.pone.0260988)
Supplement: S5 Table — (PDF) [file pone.0260988.s010.pdf]

S5 Table. Progression-Free (PFS) and overall (OS) survival effects of individual cytokines for NSCLC and SCLC

	NSCLC						SCLC					
	PFS			OS			PFS			OS		
Factor	HR(95%CI)	p-value	p-adj*	HR(95%CI)	p-value	p-adj*	HR(95%CI)	p-value	p-adj*	HR(95%CI)	p-value	p-adj*
CXCL9.MIG.	1 (0.86-1.2)	0.989	0.989	1.2 (0.99-1.4)	0.057	0.309	0.9 (0.73-1.1)	0.307	0.828	1 (0.81-1.2)	0.978	0.998
EGF	1 (0.93-1.1)	0.909	0.949	1 (0.94-1.1)	0.513	0.838	1 (0.89-1.2)	0.677	0.921	0.97 (0.81-1.1)	0.704	0.947
Eotaxin	0.86 (0.65-1.1)	0.302	0.687	0.89 (0.64-1.2)	0.488	0.838	1 (0.62-1.7)	0.878	0.925	0.94 (0.48-1.8)	0.847	0.947
FGF.2	0.98 (0.81-1.2)	0.827	0.917	1.1 (0.94-1.4)	0.190	0.629	0.83 (0.66-1)	0.110	0.572	0.91 (0.74-1.1)	0.359	0.912
FLT.3L	1 (0.9-1.2)	0.687	0.871	1.1 (0.97-1.3)	0.116	0.494	0.89 (0.7-1.1)	0.350	0.828	0.84 (0.66-1.1)	0.158	0.912
Fractalkine	0.93 (0.84-1)	0.176	0.686	1 (0.89-1.2)	0.765	0.908	0.87 (0.78-0.97)	0.011	0.277	0.9 (0.8-1)	0.068	0.912
G.CSF	1 (0.95-1.1)	0.619	0.856	1 (0.95-1.1)	0.471	0.838	0.97 (0.89-1.1)	0.456	0.828	0.94 (0.85-1)	0.259	0.912
GM.CSF	0.83 (0.52-1.3)	0.457	0.811	0.017 (0-Inf)	0.995	0.995	2.9 (0.59-15)	0.189	0.808	6.4e-07 (0-Inf)	0.998	0.998
GROalpha	1.1 (0.96-1.2)	0.196	0.686	1.1 (0.95-1.2)	0.241	0.629	0.97 (0.8-1.2)	0.799	0.921	0.97 (0.76-1.2)	0.824	0.947
IFNalpha2	0.97 (0.89-1.1)	0.517	0.811	1 (0.93-1.1)	0.517	0.838	0.91 (0.78-1.1)	0.210	0.821	0.9 (0.77-1.1)	0.198	0.912
IFNgamma	0.92 (0.82-1)	0.160	0.686	1 (0.89-1.2)	0.861	0.940	1.1 (0.89-1.3)	0.410	0.828	0.97 (0.81-1.2)	0.764	0.947
IL.10	1 (0.96-1.1)	0.290	0.687	1.1 (0.96-1.2)	0.237	0.629	0.91 (0.61-1.4)	0.652	0.921	1.1 (0.74-1.7)	0.609	0.947
IL.12p40	0.92 (0.81-1)	0.219	0.686	1.1 (0.93-1.3)	0.304	0.715	0.86 (0.71-1)	0.124	0.581	0.85 (0.7-1)	0.119	0.912
IL.12p70	0.93 (0.8-1.1)	0.310	0.687	1 (0.88-1.2)	0.697	0.862	0.88 (0.72-1.1)	0.252	0.828	0.99 (0.76-1.3)	0.913	0.993
IL.13	0.94 (0.87-1)	0.150	0.686	1 (0.92-1.1)	0.892	0.940	1 (0.59-1.7)	0.997	0.997	0.71 (0.38-1.3)	0.271	0.912
IL.15	0.97 (0.82-1.1)	0.719	0.871	1 (0.86-1.3)	0.693	0.862	0.51 (0.28-0.93)	0.029	0.277	0.58 (0.29-1.2)	0.127	0.912
IL.17A	0.98 (0.86-1.1)	0.806	0.917	1.1 (0.95-1.3)	0.208	0.629	0.92 (0.57-1.5)	0.716	0.921	1.1 (0.68-1.8)	0.691	0.947
IL.17E	1 (0.95-1.1)	0.742	0.871	1 (0.95-1.1)	0.649	0.853	0.91 (0.67-1.3)	0.576	0.873	0.94 (0.65-1.4)	0.764	0.947
IL.17F	0.93 (0.85-1)	0.116	0.686	0.97 (0.88-1.1)	0.590	0.847	0.9 (0.74-1.1)	0.333	0.828	0.96 (0.76-1.2)	0.698	0.947
IL.18	0.97 (0.83-1.1)	0.723	0.871	1.1 (0.95-1.4)	0.152	0.595	1.1 (0.87-1.4)	0.365	0.828	0.96 (0.76-1.2)	0.723	0.947
IL.1alpha	0.95 (0.84-1.1)	0.371	0.726	1.1 (0.93-1.2)	0.424	0.838	1 (0.74-1.5)	0.789	0.921	1.1 (0.76-1.6)	0.624	0.947
IL.1beta	0.93 (0.82-1.1)	0.273	0.687	1.1 (0.92-1.2)	0.433	0.838	1.1 (0.9-1.2)	0.531	0.833	1 (0.84-1.2)	0.929	0.993
IL.1RA	0.97 (0.84-1.1)	0.705	0.871	1.1 (0.92-1.3)	0.348	0.743	1 (0.87-1.2)	0.769	0.921	1.1 (0.93-1.3)	0.242	0.912
IL.2	0.99 (0.82-1.2)	0.955	0.975	1 (0.86-1.3)	0.653	0.853	0.8 (0.66-0.96)	0.019	0.277	0.82 (0.67-1)	0.047	0.912
IL.22	0.94 (0.87-1)	0.118	0.686	0.95 (0.87-1)	0.235	0.629	0.86 (0.63-1.2)	0.338	0.828	0.87 (0.63-1.2)	0.402	0.912
IL.27	1.3 (1-1.6)	0.027	0.316	1.5 (1.2-1.9)	0.002	0.046	1.1 (0.94-1.3)	0.236	0.828	1 (0.88-1.2)	0.807	0.947
IL.3	0.72 (0.42-1.2)	0.236	0.686	0.95 (0.54-1.7)	0.862	0.940	0.92 (0.77-1.1)	0.344	0.828	0.96 (0.8-1.1)	0.633	0.947
IL.4	0.97 (0.83-1.1)	0.671	0.871	1 (0.86-1.2)	0.773	0.908	0.96 (0.76-1.2)	0.693	0.921	0.93 (0.71-1.2)	0.575	0.947
IL.5	0.92 (0.77-1.1)	0.332	0.687	1.1 (0.88-1.3)	0.552	0.847	1 (0.93-1.1)	0.810	0.921	0.98 (0.89-1.1)	0.703	0.947
IL.6	1.4 (1.2-1.6)	4.49E-06	2.11E-04	1.5 (1.3-1.8)	0.000	3.39E-07	0.85 (0.73-0.98)	0.028	0.277	0.88 (0.75-1)	0.109	0.912
IL.7	0.96 (0.85-1.1)	0.565	0.811	1 (0.88-1.2)	0.896	0.940	0.98 (0.74-1.3)	0.914	0.934	1.1 (0.76-1.5)	0.683	0.947
IL.8	1.2 (1-1.4)	0.019	0.316	1.3 (1.1-1.5)	0.004	0.046	1.1 (0.92-1.2)	0.407	0.828	1 (0.88-1.2)	0.806	0.947
IL.9	0.94 (0.86-1)	0.248	0.686	1 (0.89-1.1)	0.979	0.995	1.1 (0.8-1.6)	0.493	0.828	1.2 (0.74-1.8)	0.533	0.947
IP.10	1 (0.87-1.2)	0.839	0.917	1.2 (0.99-1.4)	0.066	0.309	0.77 (0.59-0.98)	0.038	0.294	0.82 (0.62-1.1)	0.164	0.912
M.CSF	1 (0.95-1.1)	0.526	0.811	1.1 (1-1.2)	0.028	0.222	1 (0.71-1.5)	0.817	0.921	0.81 (0.53-1.2)	0.315	0.912
MCP.1	1.1 (0.85-1.4)	0.520	0.811	1.3 (0.98-1.8)	0.064	0.309	1 (0.87-1.2)	0.846	0.924	1 (0.86-1.2)	0.998	0.998
MCP.3	0.9 (0.82-0.99)	0.023	0.316	0.97 (0.87-1.1)	0.595	0.847	1 (0.89-1.1)	0.886	0.925	1 (0.89-1.2)	0.827	0.947
MDC	0.94 (0.77-1.2)	0.570	0.811	0.93 (0.73-1.2)	0.577	0.847	0.66 (0.47-0.92)	0.014	0.277	0.83 (0.58-1.2)	0.318	0.912
MIP.1alpha	0.96 (0.88-1.1)	0.389	0.731	1 (0.93-1.2)	0.484	0.838	0.79 (0.63-1)	0.048	0.325	0.89 (0.68-1.2)	0.402	0.912
MIP.1beta	1.1 (0.82-1.5)	0.549	0.811	1.2 (0.9-1.7)	0.204	0.629	0.94 (0.82-1.1)	0.413	0.828	0.95 (0.82-1.1)	0.544	0.947
PDGF.AA	1 (0.93-1.2)	0.491	0.811	1.1 (0.95-1.2)	0.256	0.633	0.57 (0.3-1.1)	0.100	0.572	0.71 (0.35-1.4)	0.340	0.912
PDGF.AB.BB	1.1 (0.95-1.2)	0.234	0.686	1.1 (0.93-1.3)	0.331	0.741	0.98 (0.81-1.2)	0.823	0.921	1.1 (0.84-1.3)	0.643	0.947
sCD40L	1.1 (1-1.2)	0.035	0.329	1.2 (1.1-1.3)	0.004	0.046	1.1 (0.88-1.3)	0.494	0.828	1.1 (0.89-1.4)	0.326	0.912
TGFalpha	0.91 (0.79-1.1)	0.230	0.686	1 (0.87-1.2)	0.900	0.940	1.1 (0.8-1.6)	0.447	0.828	1.2 (0.88-1.7)	0.249	0.912
TNFalpha	1 (0.88-1.2)	0.875	0.935	1.2 (1-1.4)	0.024	0.222	0.93 (0.76-1.2)	0.526	0.833	0.95 (0.77-1.2)	0.653	0.947
TNFbeta	0.93 (0.8-1.1)	0.336	0.687	1 (0.9-1.2)	0.621	0.853	1 (0.77-1.4)	0.788	0.921	0.82 (0.57-1.2)	0.268	0.912
VEGF.A	1.1 (0.98-1.2)	0.113	0.686	1.2 (1-1.3)	0.048	0.309	1.1 (0.9-1.3)	0.484	0.828	1.1 (0.89-1.3)	0.407	0.912

OS, Overall survival; PFS, Progression-free survival; NSCLC, Non-small cell lung cancer; SCLC, Small cell lung cancer; HR, Hazard ratio; CI, Confidence interval.

*Factor p-values were adjusted using the Benjamini-Hochberg method, and Factors with an adjusted p-value less than 0.05 are highlighted in orange.
