# Supplementary material for: Diversity and heterogeneity of immune states in non-small cell lung cancer and small cell lung cancer
Source: PLoS One. 2021 Dec 2;16(12):e0260988. doi: 10.1371/journal.pone.0260988 (PMC8638918; doi:10.1371/journal.pone.0260988)
Supplement: S6 Table — (PDF) [file pone.0260988.s011.pdf]

S6 Table. Kruskal-Wallis test results comparing each plasma factor between the four IL-6/NLR groups with multi-sample adjustment using Benjamini-Hochberg method.

Factor	p-value	adj. p-value
IL6	2.80E-12	1.32E-10
IL8	0.00051	0.012
TNFA	0.0024	0.038
IL27	0.0038	0.045
GROA	0.0088	0.083
IP10	0.013	0.102
CXCL9.MIG	0.022	0.103
MCSF	0.018	0.103
MDC	0.02	0.103
TNFB	0.021	0.103
FLT3L	0.035	0.124
IL10	0.036	0.124
IL12P40	0.036	0.124
MIP1B	0.037	0.124
IL15	0.046	0.144
GCSF	0.05	0.147
IL4	0.056	0.155
IL5	0.091	0.238
EGF	0.12	0.282
sCD40L	0.12	0.282
IL1A	0.19	0.425
IFNA2	0.35	0.47
IL1B	0.34	0.47
IL1RA	0.3	0.47
IL2	0.25	0.47
MIP1A	0.25	0.47
IL12P70	0.37	0.47
IL13	0.37	0.47
IL17A	0.31	0.47
IL17E	0.27	0.47
IL17F	0.23	0.47
IL18	0.33	0.47
IL7	0.28	0.47
IL9	0.35	0.47
PDGFAA	0.29	0.47
TGFA	0.33	0.47
VEGFA	0.25	0.47
Eotaxin	0.42	0.493
IFNG	0.42	0.493
IL3	0.43	0.493
PDGFABBB	0.41	0.493
MCP1	0.49	0.548
GMCSF	0.58	0.634
MCP3	0.71	0.758
FGF2	0.76	0.794
Fractalkine	0.85	0.86
IL22	0.86	0.86
